# Effect of vaccination route (intradermal vs. intramuscular) against porcine reproductive and respiratory syndrome using a modified live vaccine on systemic and mucosal immune response and virus transmission in pigs

**DOI:** 10.1186/s12917-023-03853-4

**Published:** 2024-01-03

**Authors:** Patricia Renson, Sophie Mahé, Mathieu Andraud, Mireille Le Dimna, Frédéric Paboeuf, Nicolas Rose, Olivier Bourry

**Affiliations:** 1 Swine Virology Immunology Unit, Ploufragan-Plouzané-Niort Laboratory, French Agency for Food, Environmental and Occupational Health and Safety (ANSES), Ploufragan, 22440 France; 2 Epidemiology, Health and Welfare Unit, Ploufragan-Plouzané-Niort Laboratory, ANSES, Ploufragan, 22440 France; 3 SPF Pig Production and Experimentation Unit, Ploufragan-Plouzané-Niort Laboratory, ANSES, Ploufragan, 22440 France

**Keywords:** PRRS virus, MLV, Immunization route, Transmission, Mucosal immune response, Vaccine efficacy, Piglets, IgA, Bronchoalveolar lavage

## Abstract

**Background:**

Porcine reproductive and respiratory syndrome (PRRS) is a viral disease with worldwide distribution and an enormous economic impact. To control PRRS virus (PRRSV) infection, modified live vaccines (MLVs) are widely used in the field, mainly administered via an intramuscular (IM) route. Currently, some MLVs are authorized for intradermal (ID) administration, which has many practical and welfare advantages. The objectives of the study were to compare the immune responses (systemic in blood and mucosal in lungs) and vaccine efficacy in preventing challenge strain transmission after IM or needle-free ID immunization of piglets with an MLV against PRRSV-1 (MLV1).

**Methods:**

Groups of sixteen 5-week-old specific pathogen-free piglets were vaccinated with Porcilis PRRS® (MSD) either by an IM (V+ IM) or ID route (V+ ID) using an IDAL®3G device or kept unvaccinated (V-). Four weeks after vaccination, in each group, 8 out of the 16 piglets were challenged intranasally with a PRRSV-1 field strain, and one day later, the inoculated pigs were mingled by direct contact with the remaining 8 sentinel noninoculated pigs to evaluate PRRSV transmission. Thus, after the challenge, each group (V+ IM, V+ ID or V-) included 8 inoculated and 8 contact piglets. During the postvaccination and postchallenge phases, PRRSV replication (RT–PCR), PRRSV-specific antibodies (ELISA IgG and IgA, virus neutralization tests) and cell-mediated immunity (ELISPOT Interferon gamma) were monitored in blood and bronchoalveolar lavages (BALs).

**Results:**

Postvaccination, vaccine viremia was lower in V+ ID pigs than in V+ IM pigs, whereas the cell-mediated immune response was detected earlier in the V+ ID group at 2 weeks postvaccination. In the BAL fluid, a very low mucosal immune response (humoral and cellular) was detected. Postchallenge, the vaccine efficacy was similar in inoculated animals with partial control of PRRSV viremia in V+ ID and V+ IM animals. In vaccinated sentinel pigs, vaccination drastically reduced PRRSV transmission with similar estimated transmission rates and latency durations for the V+ IM and V+ ID groups.

**Conclusions:**

Our results show that the tested MLV1 induced a faster cell-mediated immune response after ID immunization two weeks after vaccination but was equally efficacious after IM or ID immunization towards a challenge four weeks later. Considering the practical and welfare benefits of ID vaccination, these data further support the use of this route for PRRS MLVs.

## Background

Porcine reproductive and respiratory syndrome (PRRS) is a major concern for swine production, as the disease is endemic in most of the major pig-producing countries worldwide. PRRS virus (PRRSV) infection is mainly characterized by reproductive disorders in sows and growth retardation and respiratory troubles in growing pigs [1]. PRRS viruses, members of the *Arteriviridae* family, are divided into *Betaarterivirus suid 1* (PRRSV-1) species of European origin and *Betaarterivirus suid 2* (PRRSV-2) species originating from North America, which are now both circulating globally (www.ictv.global/taxonomy) [2]. To decrease the clinical impact of PRRSV infection and control the within-herd dynamics of infection, modified live vaccines (MLVs) directed against each PRRSV species (MLV1 for PRRSV-1 and MLV2 for PRRSV-2) are widely used in the field both in sows and in growing pigs. Most PRRS MLVs are licensed for intramuscular (IM) injection, but an increasing number of them are now also approved for intradermal (ID) administration. ID vaccination has several advantages over IM vaccination, both for animals and farmers. For the pigs, ID vaccination has benefits for both health and welfare [3]. Indeed, compared to IM immunization, ID vaccination using a needle-free injector device is less stressful and decreases the risk of MLV spread and the hazard of iatrogenic transmission of pathogens as well as needle-induced injection-site lesions [4, 5]. For farmers, ID vaccination is easy to use, rapid and safe with no risk of self-injection [6], making ID vaccination particularly suitable for PRRS stabilization programs using mass vaccination. From an immunological perspective, ID vaccination also seems particularly interesting since the dermis is rich in antigen-presenting cells, suggesting that delivery of vaccines to this layer, rather than to muscle, should be more efficient in inducing protective immune responses [7]. This favourable immune environment in the dermis can also allow antigen dose sparing [8]. Even if still debated [9], ID vaccination may also induce fully functional tissue-resident memory T cells at the local site of immunization [10], which could be of interest for PRRSV, given the mucosal portal of entry of this virus and the lung being the primary site for viral replication. The antibody response at the mucosal level is also of interest in controlling PRRSV infection. Mucosal IgA could have an important role in the control of PRRSV infection, as associations have been demonstrated between mucosal IgA response induction and the cessation of virus excretion in oral fluid samples of PRRS-unstable herds [11], as well as the strongest reduction in PRRSV replication in porcine macrophages [12].

The effect of vaccination route for PRRS MLVs has already been studied to some extent. Martelli et al. showed that ID vaccination was equally efficacious to IM vaccination in controlling both PRRSV-1-induced clinical signs and PRRSV viremia [13, 14]. Further studies from the same group also demonstrated that the systemic immune response induced after ID vaccination was at least comparable to that induced by IM immunization [15]. More recently, Aguirre et al. showed equivalent immunization induced by IM or ID administration using another MLV against PRRSV-1 [16], and similar protection was obtained for IM and ID vaccination routes using a new vaccine candidate against highly pathogenic PRRSV-2 [17]. Other studies tended to demonstrate better efficacy or at least better induction of postvaccine cell-mediated immunity for the ID route [5, 18, 19]. Nevertheless, to date, existing studies have only focused on systemic immunity, and none of them has explored the mucosal immune response, which could be a key element in preventing PRRSV infection at the point of entry. Furthermore, to our knowledge, no data are available about the vaccine efficacy on direct horizontal transmission of a heterologous PRRSV strain to vaccinated sentinel pigs after using a PRRS MLV by the ID or IM route. In this context, we proposed here (i) to evaluate the systemic and mucosal immune response in lungs after administering a PRRS MLV1 via an IM or ID route and (ii) to assess the impact of the immunization route on the efficacy of the vaccine to prevent PRRSV transmission in vaccinated sentinel pigs.

## Results

### Intradermal vaccination is associated with lower vaccine viremia and earlier induction of cell-mediated immunity

**Fig. 1:**
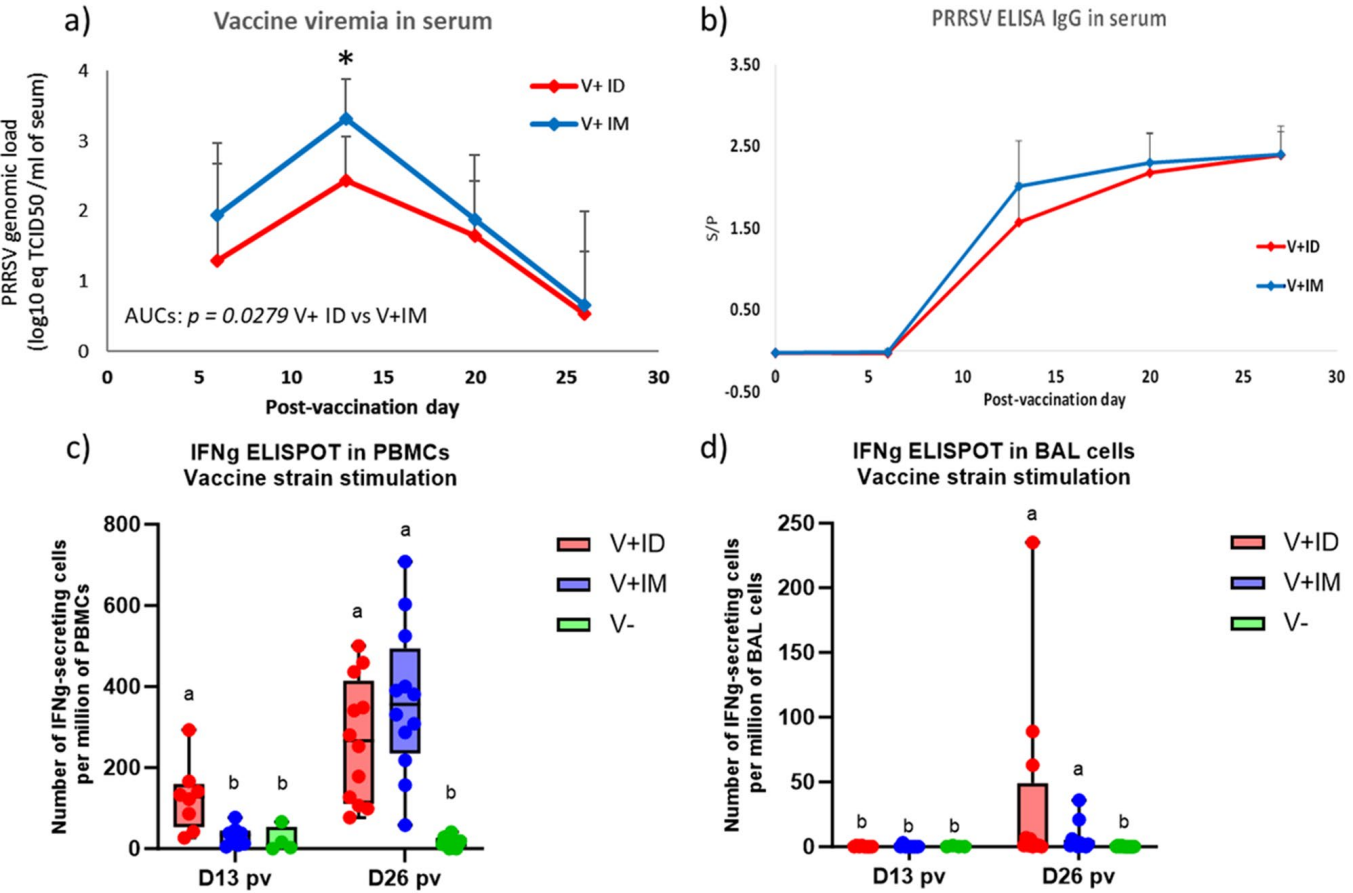
Postvaccination data. (**a**) Quantification of vaccine strain genome load by qRT–PCR in the serum of vaccinated pigs (mean + SD; 16 pigs per group). In the graph, * indicates that the V + ID group is significantly different from the V+ IM group at D13 PV with p < 0.05. (**b**) Detection of PRRSV IgG antibodies by ELISA in the serum of vaccinated pigs (mean + SD; 16 pigs per group). Count of interferon gamma-secreting cells (IFNg-SCs) responding to in vitro vaccine strain stimulation among PBMCs purified from blood (**c**) or among cells isolated from the BAL (**d**) of vaccinated and unvaccinated pigs (8 vaccinated pigs per group and 4 unvaccinated pigs at D13 PV, 12 pigs per group at D26 PV). On boxplots, different letters above the bars indicate that the groups are significantly different from each other with p < 0.05

During the postvaccination period, we first monitored vaccine viremia in vaccinated pigs. As shown in Fig. 1a, the vaccine viral load in the blood was consistently lower in the V+ ID group than in the V+ IM group, with a significantly lower vaccine viral load at D13 PV and a significantly reduced area under the curve (AUC) (p = 0.0279). Regarding the postvaccination immune response, the kinetics of seroconversion were somewhat slower in the V + ID group than in the V+ IM group, with a slightly lower mean S/P value in the V+ ID group at D13 PV (p = 0.055) (Fig. 1b). In contrast, at the same time point (D13 PV), cell-mediated immunity (CMI) induction was detected in peripheral blood mononuclear cells (PBMCs) of V+ ID pigs but not in those of V+ IM animals (Fig. 1c). Between D13 and D26 PV, the number of interferon gamma-secreting cells (IFNg-SCs) increased rapidly in the V+ IM pigs, so the level of PRRSV-specific CMI was comparable between the V+ ID and V+ IM groups at D26 PV.

In bronchoalveolar lavage (BAL) cells, the CMI was lower than that in PBMCs, with higher individual variability. Responder pigs could be identified only from D26 PV, with no significant difference between the V+ ID and V+ IM groups (Fig. 1d). In BAL fluid, the vaccine strain detection was low, with no significantly different viral loads quantified at D13 PV and D26 PV between the V+ ID and V+ IM groups (mean 1.07 ± 1.84 log_10_ eq TCID_50_/ml for V+ ID and 2.03 ± 0.81 log_10_ eq TCID_50_/ml for V+ IM at D13 PV and mean 0.78 ± 1.29 log_10_ eq TCID_50_/ml for V+ ID and 0.07 ± 1.09 log_10_ eq TCID_50_/ ml for V+ IM at D26 PV). Nevertheless, at D13 PV, the vaccine strain was detected in 5/8 V + ID pigs and 8/8 V+ IM pigs (data not shown). In contrast, at D26 PV (4 days before the challenge), a slightly higher proportion of PRRSV was detected in the lungs of V+ ID and V+ IM pigs (7/8 for V+ ID and 5/8 for V+ IM). Regarding the antibody response in BAL fluid, no or very low induction of both PRRSV-specific IgG and IgA was detected after vaccination (see below for details).

### Both intradermal and intramuscular vaccination provide partial control of viremia in inoculated pigs but nearly complete protection in contact animals

After challenge with the PRRSV Finistere strain, very few clinical signs were noticed in pigs regardless of the group (no respiratory symptoms were observed, and only 3 V- pigs showed hyperthermia for one day). Regarding growth parameters, the average daily weight gain (ADWG) of inoculated pigs was lower during the first 18 days postchallenge in the 3 inoculated groups (0.810, 0.685 and 0.792 kg/day for the V-, V+ ID and V+ IM groups, respectively) than in the noninoculated control group (1.001 kg/day), with significant differences for the V- and V+ ID groups (p < 0.05) and a trend for the V+ IM group (p = 0.069). In contrast, no significant difference in ADWG was shown between V-, V+ ID and V+ IM inoculated pigs. At necropsies performed from D42 to D49 PC, no macroscopic pulmonary lesions were observed.

**Fig. 2:**
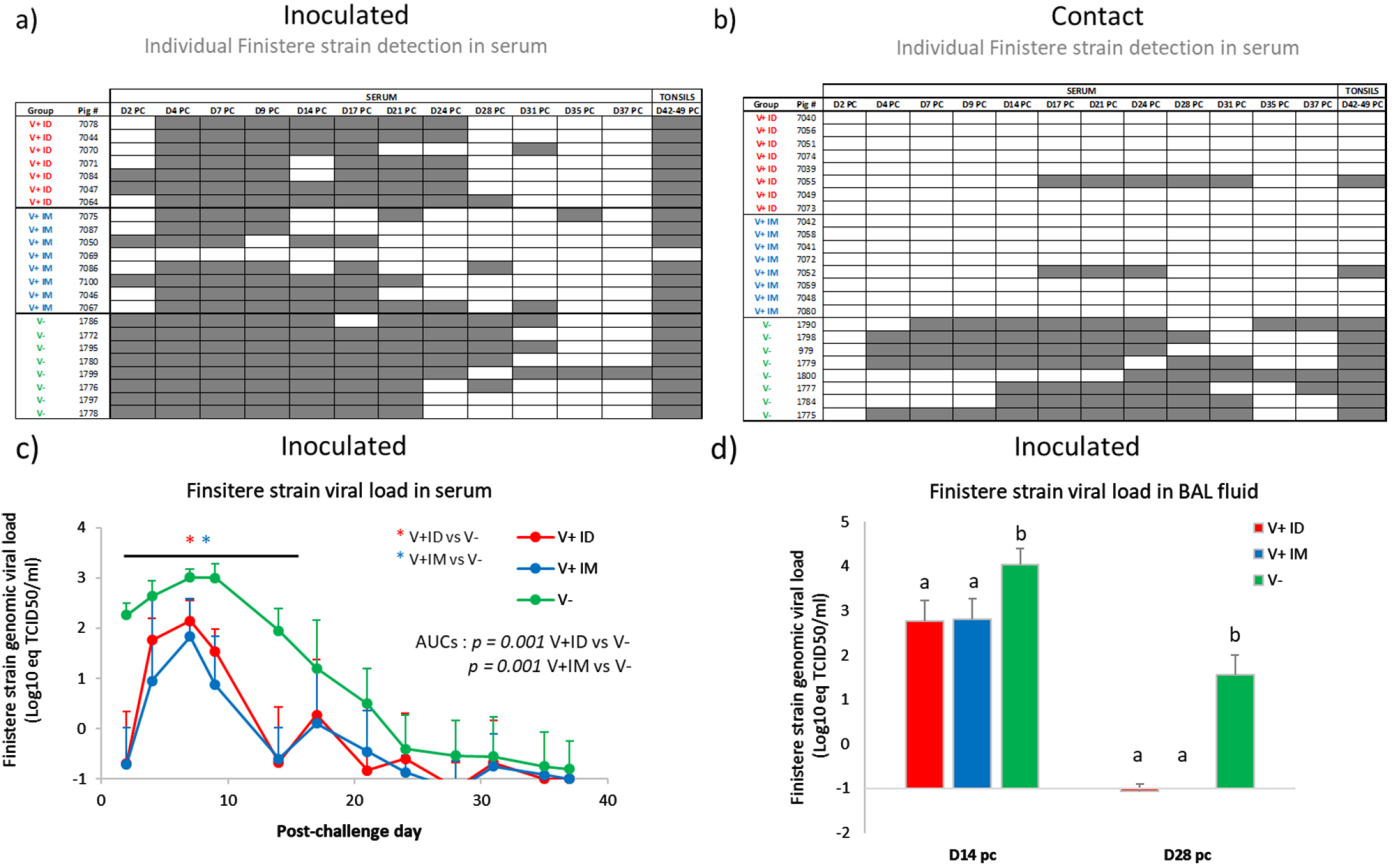
Virological data during the postchallenge period. Detection of the PRRSV Finistere strain genome by qRT–PCR for each inoculated (**a**) or contact (**b**) pig in serum and tonsils. Grey areas: positive detection; White areas: negative detection. Quantification of PRRSV Finistere strain genome load by qRT–PCR in serum (**c**) and BAL fluid (**d**) collected from inoculated and infected pigs (mean + SD; 8 pigs per group for V+ ID and V- groups and 7 pigs for V+ IM group for serum, 6 pigs per group for V+ ID and V- groups and 5 pigs for V+ IM group for BAL fluid). Red star indicates where the V+ ID group is significantly different from the V- group, and blue star indicates where the V+ IM group is significantly different from the V- group. For the histograms, different letters above the bars indicate that the groups are significantly different from each other with p < 0.05

The vaccine efficacy was more clearly evidenced by the virological data. Among the inoculated pigs, all the animals from the V+ ID group (8/8) were infected by the Finistere strain, whereas one pig in the V+ IM group (#7069) was protected from infection with no detection of the challenge strain genome either in the blood throughout the experiment or in the tonsils at necropsy (Fig. 2a). In V+ ID and V+ IM inoculated and infected pigs (excluding protected pig #7069), the Finistere strain viral load in the blood was significantly lower than in unvaccinated (V-) pigs at each time point from D2 to D14 PC, as well as for the whole kinetics assessed by AUCs, with no difference between the V+ ID and V+ IM groups (Fig. 2c). In the BAL fluid, a similar reduction in PRRSV load was observed at both D14 and D28 PC for the V+ ID and V+ IM groups relative to the V- group (Fig. 2d). In tonsils collected at necropsies, no difference in challenge strain viral loads was obtained between V+ ID and V+ IM inoculated pigs (data not shown).

Among the contact pigs, all unvaccinated pigs (V-) were infected, as demonstrated by detection of Finistere strain viremia during the follow-up period, which was then confirmed by the detection of the Finistere strain genome in the tonsils at the end of the experiment (Fig. 2b). In contrast, in V+ ID and V+ IM contact pigs, only one animal showed Finistere viremia in each group (#7055 and #7052). These results were also confirmed in the tonsils.

Using the virological data from inoculated and contact pigs from the V-, V+ ID and V+ IM groups, we then estimated the transmission parameters of the Finistere strain through mathematical modelling. As shown in Table 1, compared to that of the unvaccinated group, the latency duration was increased by one day in both vaccinated groups (2.5 for V+ ID or 2.4 for V+ IM versus 1.4 days for V-), whereas the transmission rate was markedly decreased (0.008 for V+ ID or 0.009 for V+ IM compared to 0.25 for V-).

**Table 1:** Transmission parameters

	Transmission rate		Latency (days)	
	median	95% CI*	median	95% CI
V-	0.250	[0.11; 0.50]	1.4	[0.9; 2.7]
V+ ID	0.008	[4E-4; 0.04]	2.5	[1.3; 5.6]
V+ IM	0.009	[5E-4; 0.04]	2.4	[1.3; 5.6]

*confidence interval

### A similar PRRSV immune response was induced postchallenge in intramuscularly and intradermally vaccinated pigs

Having first monitored the virological parameters, we then explored the PRRSV-specific immune response during the postchallenge period both in the blood and in the lungs.

**Fig. 3:**
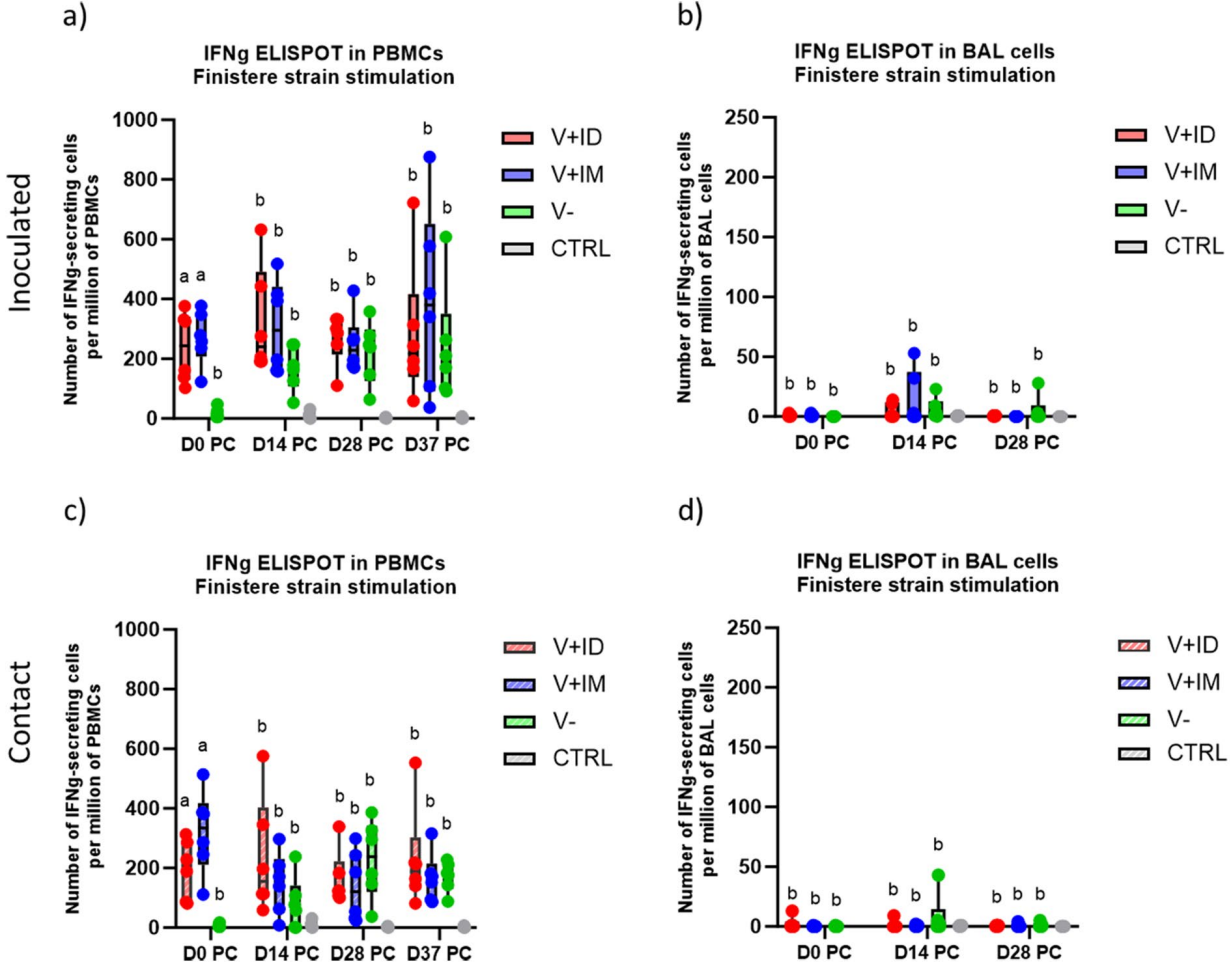
Cell-mediated immune response during the postchallenge period. Count of interferon gamma-secreting cells (IFNg-SCs) responding to in vitro Finistere strain stimulation among PBMCs purified from the blood of inoculated (**a**) or contact pigs (**c**) or among cells isolated from the BAL of inoculated (**b**) or contact pigs (**d**) (6 pigs per tested group; 3 pigs for controls). Different letters above the boxplots indicate that the groups are significantly different from each other with p < 0.05. Due to a lower number of assessed control pigs, statistical comparisons did not include the CTRL group

In the blood, the ELISPOT IFNg results showed that the Finistere strain-specific cellular response was almost stable in the V+ ID and V+ IM inoculated pigs from D0 to D37 PC (Fig. 3a). As expected, the CMI in V- pigs increased gradually after the PRRSV challenge. Similarly, the CMI seemed to be approximately stable in V+ ID and V+ IM contact pigs, whereas this response progressively increased in the V- contact pigs (Fig. 3c). In the lung, the CMI detected during the postchallenge stage towards the Finistere strain in BAL cells was very low, with no difference between the vaccinated and unvaccinated groups (Fig. 3b and d).

**Fig. 4:**
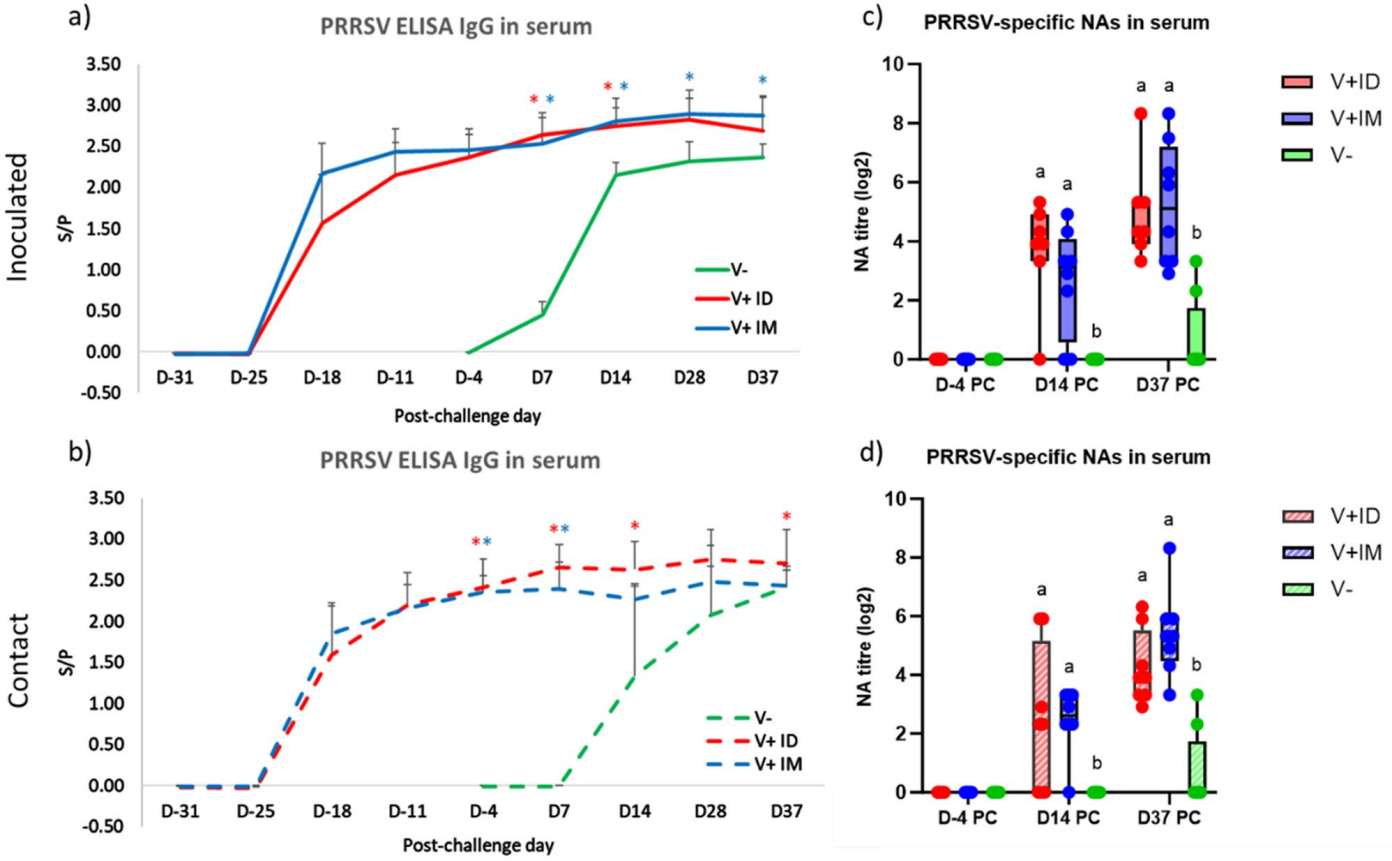
Systemic humoral response during the postchallenge period. Detection of PRRSV IgG antibodies by ELISA in the serum of inoculated (**a**) or contact pigs (**b**) (mean + SD; 8 pigs per group). Red star indicates that the V+ ID group is significantly different from the V- group, and blue star indicates that the V+ IM group is significantly different from the V- group. Detection of PRRSV neutralizing antibodies by the virus neutralizing test against the vaccine strain in the serum of inoculated (**c**) or contact pigs (**d**) (8 pigs per group). In boxplots, different letters above the bars indicate that the groups are significantly different from each other with p < 0.05

Regarding the systemic humoral response in inoculated pigs, no significant increase in the PRRSV IgG antibody level was shown in serum for the V+ IM or V+ ID groups after the challenge (Fig. 4a). For the unvaccinated inoculated pigs, 5 out of 8 animals seroconverted at D7 PC, and all (8/8) were seropositive at D14 PC. In contact pigs, as expected, seroconversion was delayed in the V- pigs (compared to the V- inoculated pigs), with 5 out 8 pigs seropositive at D14 PC and all the pigs having seroconverted at D37 PC. As in inoculated pigs, the level of anti-PRRSV IgG in serum remained stable for V+ IM and V+ ID contact pigs during the postchallenge period (Fig. 4b).

Regarding neutralizing antibodies (NAs) in the blood, no neutralizing activity directed against the vaccine strain was detected before challenge (D-4 PC) in either vaccinated group (Fig. 4c and d). At D14 PC, NAs were detected in both inoculated and contact pigs for the V+ IM and V+ ID groups, with no difference between groups according to the vaccination route. Then, at D37 PC, the level of NAs increased for all vaccinated pigs, without a quantitative difference between inoculated and contact pigs. In unvaccinated (V-) pigs, NAs were detected late (at D37 PC) and only in 2 out of 8 inoculated and 1 out 8 contact pigs.

**Fig. 5:**
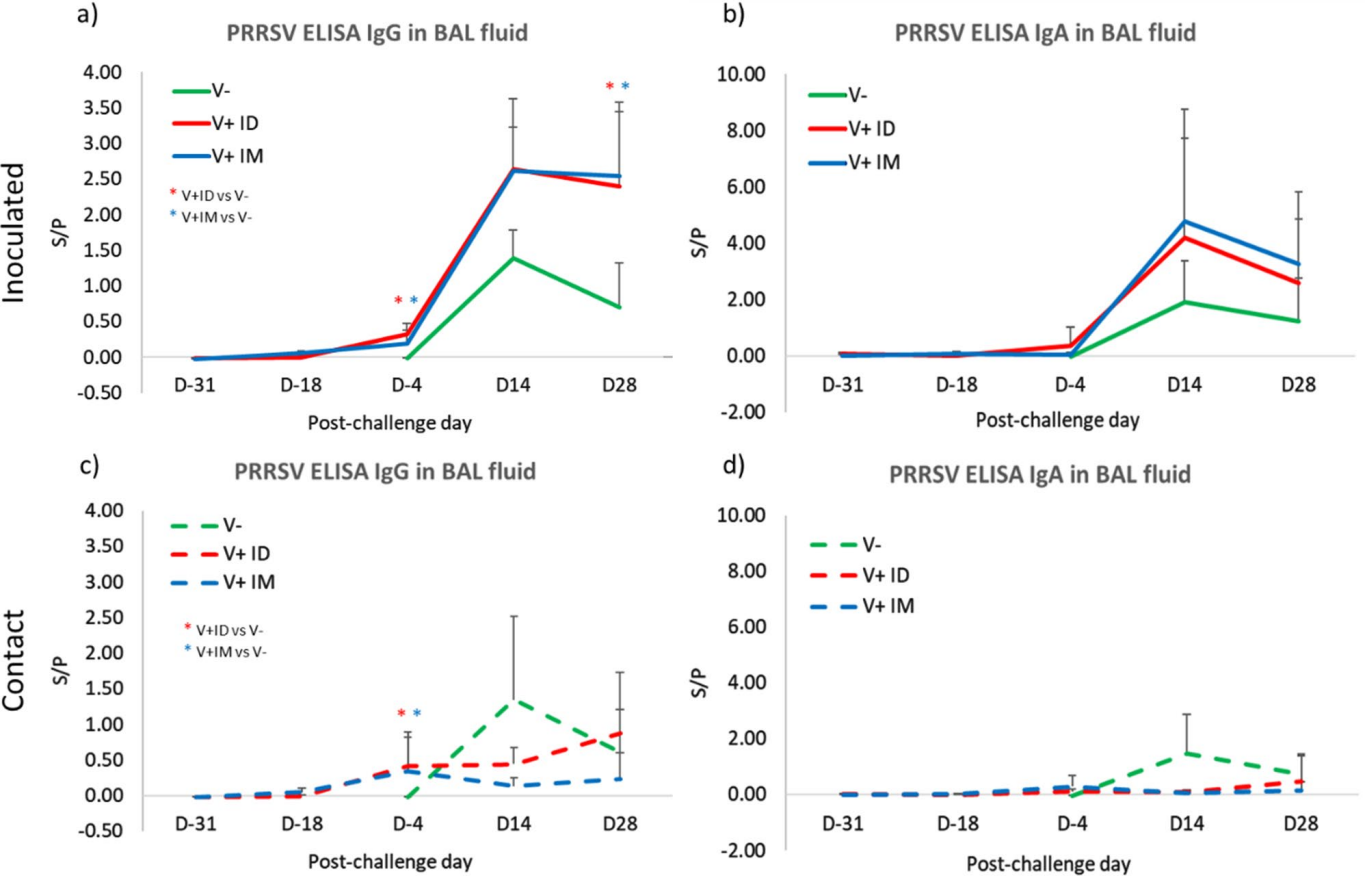
Mucosal humoral response during the postchallenge period. Detection of PRRSV IgG antibodies by ELISA in BAL fluid of inoculated (**a**) or contact pigs (**c**) (mean + SD; 6 pigs per group). Detection of PRRSV IgA antibodies by ELISA in BAL fluid of inoculated (**b**) or contact pigs (**d**) (mean + SD; 6 pigs per group). The red star * indicates that the V + ID group is significantly different from the V- group, and the blue star * indicates that the V + IM group is significantly different from the V- group

At the lung level, in the V+ IM and V+ ID groups, no or only very low levels of IgG antibodies were detected after vaccination (between D-31 to D-4 PC), both in future inoculated or contact pigs (Fig. 5a and c). In inoculated pigs, the detection of anti-PRRSV antibodies mainly occurred after the Finistere challenge in both V+ IM and V+ ID pigs, with IgG antibody levels significantly higher than those in V- inoculated pigs at D28 PC (Fig. 5a). In the contact pigs (Fig. 5c), the levels of IgG were stably low for V+ IM pigs (1/6 seropositive pigs from D-4 to D28 PC) but increased progressively for V+ ID pigs after the challenge (4/6 seropositive pigs at D14 PC); nevertheless, there was no significant difference in antibody level between the 2 groups. In the V- contacts pigs, the kinetics of the IgG S/P value in BAL fluid resembled that of V- inoculated pigs with 4/6 positive pigs at D14 PC.

Regarding the detection of IgA in the lungs, the picture was close to what was seen for IgG, with no or very low antibody detection before the challenge and detection of anti-PRRSV antibodies at D14 PC in both inoculated pigs (regardless of vaccination) and unvaccinated contacts (Fig. 5b and d). In the contact, vaccinated pigs, PRRSV-specific IgA was detected only in the pigs infected by the Finistere strain (V+ IM pig #7052 and V+ ID pig #7055). Finally, we were not able to detect PRRSV-specific NAs in the BAL fluid, regardless of group (data not shown).

## Discussion

In the first part of this study, we explored vaccine replication and the vaccine strain-specific immune response in piglets immunized via an IM or ID route, both at the systemic (blood) and mucosal (lung) levels. Two weeks after vaccination, we showed that for V+ ID piglets, decreased vaccine viremia was associated with an earlier induction of CMI. First, the virological and immunological results seem globally coherent together. Indeed, we can speculate that the enhanced CMI against the vaccine strain in V+ ID pigs may have led to better control of vaccine strain replication within the first two weeks postvaccination, as IFNg is known to inhibit PRRSV replication [20, 21]. Second, the data from the postvaccination follow-up also seem consistent with the results of previous studies. Using another commercial MLV1, an earlier and stronger CMI induction was described by Madapong et al. in ID-vaccinated pigs than in IM-vaccinated pigs before the challenge [22]. Similarly, using a commercial MLV2, Madapong et al. showed lower vaccine viremia at D14 PV, earlier seroconversion at D21 PV and a stronger CMI for the ID route at D35 and D42 PV than for the IM vaccination route [5], suggesting that the enhanced immunogenicity of ID immunization might be extended to different PRRS MLVs. However, other authors either showed a similar IFNg response in the postvaccination period between ID- and IM-vaccinated pigs using the same MLV1 we used [14, 15] or reported a significantly higher IFNg response in piglets vaccinated by the IM route than in those vaccinated by the ID route at D35 PV but not at D42 PV using another MLV1 [16]. In our study, from the third week postvaccination, equivalent results were obtained between the two vaccination routes. The stimulation of the cell-mediated immune response by ID vaccination could be dependent on the vaccine used and on individual host factors. The earlier induction of CMI by the ID route observed in this study must be confirmed and recorded at more than one time point to support the hypothesis that it could induce better protection against an early challenge occurring around the second week after vaccination.

In the lung, during the postvaccination phase, the PRRSV-specific immune response we measured at both the humoral and cellular levels was very low. These results are consistent with our previous results for very weak CMI induction [23] and with those of Toman et al., who also showed a very low induction of IgG and IgA in the BAL fluid of pigs vaccinated with the same vaccine by the IM route [24]. This delayed immune response could be linked to the later (and probably lower) lung distribution of PRRSV MLV strains compared with field strains. Indeed, compared with the serum, we previously described a delay for vaccine strain detection (from D10 PV) and consequently for IgG detection (from D21 PV) in BAL fluid from IM-vaccinated pigs (using the same MLV1 used in the present study) [23]. In contrast, concomitant IgG detection in serum and BAL fluid (from D7-9 postinoculation) was observed for animals inoculated intranasally with a field PRRSV-1 strain [25].

Our results showed similar MLV1 strain detection in BAL fluid for IM- and ID-vaccinated pigs, whereas higher and longer vaccine strain persistence was observed by Madapong et al. in nasal swabs and BAL fluid from MLV2 IM-vaccinated pigs (relative to ID), suggesting a lower risk of vaccine strain transmission for ID vaccination [5]. Therefore, even if we detected lower vaccine viremia for the ID route, we cannot truly assume that for the MLV1 we used in the present study, the ID immunization route may truly minimize vaccine transmission.

In the second part of the study, we evaluated the efficacy of the PRRS MLV1 vaccine (delivered via an IM or ID route) towards a challenge occurring 4 weeks after vaccination with the low virulence Finistere PRRSV-1 strain, which mostly limited the comparison to virological parameters. In our SPF pig PRRSV-1 mono-infection model, clinical signs are indeed scarce, even when inoculating the higher virulent recombinant Horsen strain that induced reproductive failures and high piglet mortality in Danish herds [26]. As expected, and consistent with our previous studies using the same vaccine and challenge strain [27, 28], we demonstrated decreased viremia in inoculated pigs for both vaccinated groups, with no significant difference between vaccination routes. Surprisingly, one pig in the V+ IM group (#7069) was fully protected from infection. As the PRRSV-specific response of this pig was not particularly high, we have no concrete explanation for this full protection.

Given the similar immune response levels measured in V+ ID and V+ IM vaccinated pigs at the time of the challenge, the absence of a difference in vaccine protection between immunization routes was predictable. These results are comparable to those of Martelli et al. [13] and Jiang et al. [17], who previously showed, in a homologous MLV1/PRRSV-1 and MLV2/PRRSV-2 challenge model, the same partial viremia control in pigs vaccinated by IM or ID routes, respectively, and no effect of the immunization route. In contrast, Madapong et al. [22] showed in a heterologous MLV1/PRRSV-2 challenge model that ID-vaccinated pigs had significantly lower PRRSV viremia and lung lesion scores than IM-vaccinated pigs, but in their case, a higher CMI was induced by ID vaccination and maintained until the time of the challenge.

During the postchallenge phase, we monitored the PRRSV-specific immune response in both the blood and lungs. At the systemic and group levels, no measurable booster effect was observed in either the cell-mediated or humoral immune response after challenge in vaccinated animals. Nevertheless, at the individual level, a booster effect in the IFNg-SC response can be observed in half of IM- or ID-vaccinated and inoculated piglets. In the other pigs, the absence of a booster effect could globally be related to lower challenge strain viral loads or, in one case, the total absence of virus detection in serum.

As shown by Madapong et al. [22], no anamnestic response was detected in the vaccinated and inoculated pigs according to the IgG ELISA. These results are somewhat different from those of Ferrari et al. [15], who showed a clear increase in antibody levels after exposure to the challenge strain. This discrepancy between the two studies may be linked to the low antibody level detected after vaccination by Ferrari et al. Commercial anti-PRRSV ELISAs are not quantitative methods, and our results supposed that the assessed sera were already at saturation levels at the time of the challenge. However, the NA levels measured in the vaccinated inoculated pigs were similar to those quantified in contact-vaccinated pigs, which were not infected, supporting the absence of an anamnestic humoral response after the vaccinated pigs were infected with the Finistere strain. Globally, this study showed good vaccine protection corresponding to a good reduction in PRRSV replication and transmission that was insufficient to restimulate systemic immune responses in many piglets. In the lung, the levels of IgG and IgA were very low after vaccination but increased rapidly after challenge in the inoculated pigs. In contrast, in unvaccinated pigs, we detected a quick rise in IgG and IgA levels after inoculation. These data suggest that the vaccine strain that does not easily replicate in the lung [29] could not induce a mucosal immune response of the same strength as a wild-type strain but could nevertheless prime the PRRSV-specific immune response at this site.

Our study was the first to evaluate the effect of the PRRS MLV1 immunization route on the transmission of a PRRSV-1 field strain. Among vaccinated contact pigs, we demonstrated a strong reduction in PRRSV-1 transmission, with no difference between the V+ ID and V+ IM groups. These results are completely in line with our previous study [28] with transmission parameters that are very close between the two studies. The absence of a difference between vaccination routes in preventing transmission of the challenge strain to contact pigs is consistent with the challenge strain viral loads quantified in serum, BAL fluid, and tonsils, which were not different between the V+ ID and V+ IM inoculated groups. Furthermore, our results are in agreement with those of Dortmans et al. [30], who previously evaluated the effect of vaccination route on classical swine fever virus (CSFV) spread. In this case, they showed that ID vaccination does not result in better protection against horizontal transmission of CSFV compared to IM vaccination.

Having monitored the immune response both locally and systemically, it might be attractive to attempt to identify protective correlates in these contact animals that have been mostly protected. In the serum as in the BAL fluid, no NAs were detected before the challenge in the vaccinated pigs. Therefore, although NAs had been previously shown to be able to protect against PRRSV infection [31], our data could not confirm the role of NAs in the protection of vaccinated contact pigs. If we consider more specifically that the mucosal immune response might be the first line of defence against PRRSV, we can see that the 2 unprotected pigs (#7055 and #7052) among the vaccinated contact animals had higher IgG levels in the BAL fluid at the time of challenge (data not shown). As recently reported by Ruggeri et al. [12], these unexpected results might suggest a possible involvement of antibody-dependent enhancement of PRRSV transmission linked to PRRSV-specific mucosal IgG.

## Conclusion

Our results showed that despite an earlier induction of CMI for the V+ ID group, the vaccine efficacy was equivalent for both immunization routes, certainly due to the equivalent immune responses present at the time of challenge (4 weeks after vaccination). This equivalent efficacy between ID and IM vaccination is nonetheless interesting considering all the other advantages of the former in terms of animal welfare, the lower risk of pathogen transmission, the rapidity of use and user comfort. Considering the strong reduction in PRRSV-1 transmission in ID-vaccinated pigs, our study results reinforce the interest in ID mass vaccination in cases of PRRSV stabilization protocols where a strong effect of the vaccine on PRRSV transmission is essential.

## Methods

### Viruses and vaccine

The Porcilis PRRS vaccine (MSD, Beaucouzé, France; batch No. A213BB01) was used for the animal experiment as an MLV1 vaccine (for delivery via intradermal and intramuscular routes). According to the manufacturer product sheet, regardless of the route, one vaccine dose corresponds to 10^4.0^ to 10^6.3^ TCID50 of live virus depending on the vaccine batch. As we used the same vaccine batch for IM and ID vaccination, all the animals received the same vaccine titre. For in vitro analyses, the MLV1 vaccine strain (DV strain) was obtained by suspending the lyophilized vaccine in Eagle’s minimal essential medium (EMEM), propagating and titrating it on MARC-145 cells for two and three passages for virus neutralization tests and ELISPOT analyses, respectively.

The so-called PRRSV-1 “Finistere” strain, belonging to subtype 1 and referenced as PRRS-FR-2005-29-24-1 (GenBank accession No. KY366411), was used for the animal challenge. This field strain, isolated in France in 2005 from a sow in a herd with reproductive failures, is representative of the local strains circulating in the area but shows a low virulence in SPF piglets [32]. The “Finistere” strain was propagated and titrated in porcine primary alveolar macrophages for two passages for animal inoculation and for six passages for ELISPOT analyses.

The “Finistere” strain and the DV vaccine strain, both PRRSV-1 subtype 1 strains, shared 92.41% and 92.56% nucleotide sequence identity on ORF5 and the full genome, respectively.

### Experimental settings

**Fig. 6:**
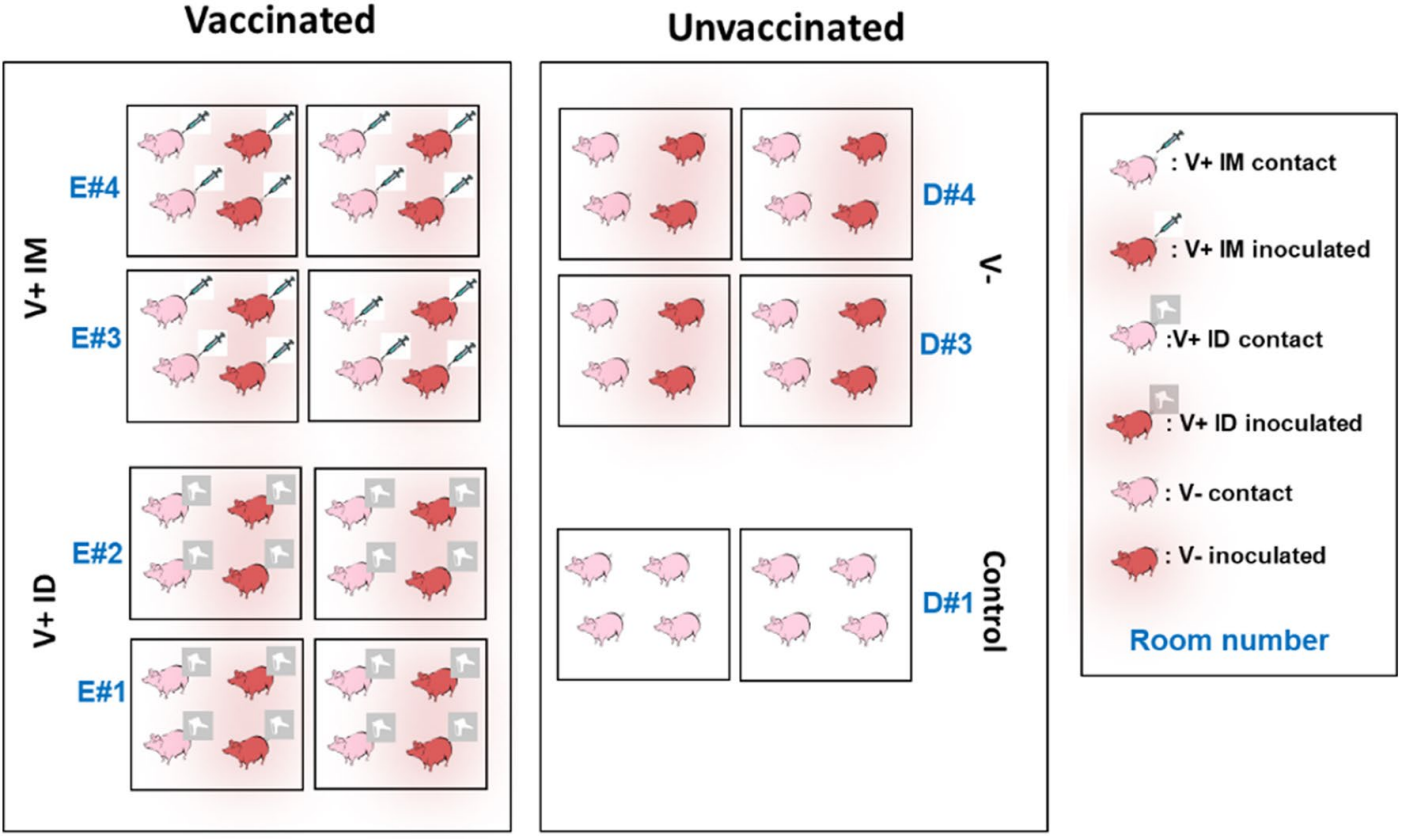
Experimental design

The experiment was carried out in our protected animal facilities in Anses-Ploufragan using Large White Specific Pathogen Free (SPF) piglets produced in Anses or in the “Centre technique des productions animales et agroalimentaires” in Ploufragan. Fifty-six 5-week-old piglets were included in the experiment and randomly distributed into 7 independent rooms stratified by weight and sex (8 pigs per group, corresponding to the minimum number of animals necessary to observe significance according to the t–test power calculation). Rooms E#1 and E#2 housed intradermally vaccinated pigs (V+ ID group; 7.2 +/-1.6 kg at inclusion; 8 females and 8 males), E#3 and E#4 housed intramuscularly vaccinated pigs (V+ IM group; 7.2 +/- 1.7 kg; 8 females and 8 males), D#3 and D#4 contained nonvaccinated pigs (V- group; 7.8 +/-1.1 kg; 4 females and 12 males) and D#1 housed the control pigs (7.2 +/-1.0 kg; 5 females and 3 males) (Fig. 6). In each room, two pens (50 cm apart) were separated by a solid partition and, together with air extractions above the pens, prevented the transfer of infectious material from one pen to another. Each pen included 2 inoculated and 2 contact pigs. The day of vaccination (D0 postvaccination (PV)), pigs in the V+ IM group received one dose (2 ml/pig) of Porcilis PRRS® (resuspended in Diluvac®) by an intramuscular route in the neck according to the manufacturer’s instructions. In the same way, pigs in the V+ ID group received the same antigen dose (0.2 ml/pig) of the same vaccine batch through an intradermal route using an IDAL® 3G system, specifically designed for this vaccine administration route.

The day of the challenge (D0 postchallenge (PC)), that is, at D31 PV, 2 pigs per pen were inoculated intranasally with the Finistere PRRSV strain. The inoculated piglets received 5 × 10^5^ TCID_50_ of the inoculum (2.5 ml per nostril). The piglets to be inoculated were randomly chosen in each pen and grouped together within a pen for inoculation and then distributed with their corresponding contact penmates 24 h postinoculation.

During the postvaccination period, blood samples were collected (without anticoagulant) once a week to monitor vaccine replication and the PRRSV-specific humoral immune response. At D13 and D26 PV, additional heparinized blood samplings and bronchoalveolar lavages (BALs) were performed to assess the PRRSV-specific cellular immune response both in the blood and in the lung and to detect the vaccine strain in the lung. BAL was performed under general anaesthesia to avoid pain following the intramuscular injection of 10 mg/kg Zoletil (Virbac, Carros, France) by infusing 2 × 20 ml of sterile phosphate buffered saline (PBS) using a tracheal probe (Vygon, Ecouen, France) whose size and diameter depended on the age and weight of the animal. Because the lengthy procedure for collecting BAL could affect the cell viability for performing ELISPOT on fresh cells, BAL and reciprocal heparinized blood sampling could not be applied to all animals. During the postvaccination period, heparinized blood and BAL samples were collected from 8 animals in the V+ ID and V+ IM groups and from 4 V- animals. Postchallenge, heparinized blood and BAL samples were collected from 12 animals in the V+ ID, V+ IM and V- groups (6 inoculated and 6 contact) and from 4 animals in the control group.

Cells from the BAL were separated from the fluid by centrifugation at 400 × g for 10 min, and serum was separated from whole blood by centrifugation at 3500 × g for 5 min.

After the challenge, rectal temperatures and clinical signs were recorded daily using a template adapted from Weesendorp et al. [33]. Temperatures higher than 40 °C were reported as hyperthermia. To evaluate growth performance, the animals were weighed once a week during the experiment (twice a week during the two first weeks PC), and the average daily weight gain of each animal was calculated. Blood was collected twice a week and BAL at D14 and D28 PC to quantify Finistere strain viremia and to evaluate the immune responses. All animals were euthanized and necropsied between D42 and D46 PC for macroscopic lesion observations as well as to collect tonsils for PRRSV detection.

Euthanasia was performed by bleeding the animals, which had been anesthetized by intravenous injection of Zoletil 100 (10 mg/kg). This procedure was approved by the veterinarian and the animal welfare officer of Anses Ploufragan-Plouzané-Niort laboratory.

### RT‒PCR

The PRRSV RNA genome was purified from serum and BAL fluid using NucleoSpin 8 virus kits according to the manufacturer’s instructions (Macherey-Nagel, Düren, Germany). During the postvaccination period (before the challenge), the vaccine strain genome was quantified in serum using in-house panPRRSV-1 ORF7 qRT‒PCR. After the challenge, the Finistere strain genome was specifically detected in serum, BAL fluid or tonsil homogenate by in-house ORF5 qRT‒PCR.

Briefly, PRRSV genomic RNA was amplified using the SuperScript III platinum one-step qRT‒PCR kit (Life Technologies, Carlsbad, CA, USA) with probes and primers targeting either ORF7 of the PRRSV-1 strains (forward primer, 5’- AACGYTCCCTCTGCTTGC-3’, reverse primer, 5’-CTCAACCTGAAAACTGACCTTCC-3’, probe, 5’-6FAM-CGATCCAGACGGCTTTYAATCAAGGCG-TAM-3’) or ORF5 of the Finistere strain (forward primer, 5’-TATGCGAGCTGAATGGGACC-3’, reverse primer, 5’-AGGATATGAGTGGCAACCGG-3’, probe 5’-6FAM-TGGGCAGTTGAGACTTTCGTGCT-TAM-3’). Both PRRSV RT–PCRs were conducted in duplex with the amplification of the porcine beta-actin gene as the internal control (forward primer, 5’-CTCGATCATGAAGTGCGACGT-3’, reverse primer 5’-GTGATCTCCTTCTGCATCCTGTC-3’, probe 5’-TET-ATCAGGAAGGACCTCTACGCCAACACGG-BHQ1-3’). qRT‒PCR was performed on a Chromo4 real-time PCR device (Bio-Rad, Hercules, CA, USA) using the following program: 50 °C for 30 min, 94 °C for 2 min, 45 cycles each of 94 °C for 15 s and 60 °C for 30 s. The PRRSV genomic loads in sera and BALF samples were quantified using a standard viral range of either the DV vaccine strain or the Finistere strain (with known infectious titres) diluted in the corresponding biological matrix collected from SPF pigs. The results were expressed as equivalent (eq) TCID50/mL of the type of sample used.

### ELISPOT

From heparinized blood, PRRSV-specific interferon gamma-secreting cells (IFNg-SCs) were quantified in triplicate from fresh PBMCs and purified by Ficoll-Paque™ Plus (Cytiva, Marlborough, MA, USA) density gradient centrifugation with LeucoSep tubes (Greiner Bio One, Les Ulis, France) using 16 h PRRSV stimulation of 4 × 10^5^ cells with a multiplicity of infection of 0.2 for either the vaccine strain or the Finistere strain (obtained as described above). From the BAL, a similar protocol was used to quantify IFNg-SCs from freshly purified BAL cells, using 5 × 10^5^ cells as a unique modification. In addition, each sample was stimulated with culture media or 10 µg/ml PHA (Eurobio, Les Ulis, France) as controls. PBMCs or BAL cells were stimulated on MultiScreen-IP, 0.45 μm nitrocellulose plates (reference MAIPS4510, Millipore, Burlington, MA, USA) previously coated overnight at 4 °C with 500 ng/well of an anti-pig IFNg monoclonal antibody (mAb) (clone P2G10, BD Biosciences, San Jose, CA, USA). After cell stimulation, secreted IFNg was visualized by incubating the plates for 2 h at room temperature (RT) with 25 ng/well of a biotinylated anti-pig IFNg mAb (clone P2C11, BD Biosciences, San Jose, CA, USA), followed by incubation for 1 h at RT in streptavidin alkaline phosphatase (1:1000 dilution, Caltag Medsystems, Buckingham, UK) and then for 20 min at RT in alkaline phosphatase substrate kit reagents (Bio-Rad, Hercules, CA, USA). The number of spots per well was counted using an ImmunoSpot S6 UV Analyzer (CTL, Shaker Heights, OH, USA). The results are reported as the number of IFNg-SCs per million PBMCs or BAL cells.

### ELISA

Immunoglobulin (Ig) G against PRRSV was detected in serum using PRRS X3 Ab ELISA tests according to the manufacturer’s protocol (IDEXX laboratories, Liebefeld, Switzerland). Sample-to-positive (S/P) ratios with values equal to or greater than 0.4 were considered positive according to the kit instructions.

For anti-PRRSV IgG detection in BAL fluid, the components of the commercial PRRS X3 Ab ELISA kit were used following the manufacturer’s protocol developed for serum, only modifying the starting dilution from 1:40 to 1:2.

For anti-PRRSV IgA detection, the components of the commercial PRRS X3 Ab ELISA kit were used following the manufacturer’s protocol, either only replacing the conjugated antibody of the kit by a goat anti-pig IgA HRP (Bethy, Montgomery, TX, USA) used at 1:3000 dilution in the kit diluent solution for IgA detection in serum, as described by Rotolo et al. [34], or both modifying the starting dilution to 1:2 and replacing the conjugated antibody by the goat anti-pig IgA HRP for IgA detection in BAL fluid.

In addition, for IgA and IgG assays from BAL fluid, the negative and positive controls included in the Idexx PRRS X3 Ab ELISA kit were replaced by in-house controls made in BAL fluid, calibrated as those from the kit to calculate sample-to-positive (S/P) ratios.

### Virus neutralization test

PRRSV-specific neutralizing antibodies (NAs) targeting the vaccine strain were quantified in serum using MARC145 cells. Heat-inactivated sera were two-fold serially diluted at 56 °C for 30 min, and then 50 µl of each dilution was incubated in duplicate in 96-well microtiter plates with the MLV1 DV strain at 10^1 ± 0.5^ TCID_50_/50 µl for 1 h at 37 °C with rocking agitation. A suspension of MARC-145 cells (0.5 × 10^5^ per well) was then added. After incubation for five days at 37 °C, the titres were determined using Karber’s method as the reciprocal of the highest dilution of serum that prevented virus infection of the cell monolayer, as determined by the absence of cytopathic effects in half of the duplicate wells. The neutralizing titres were expressed in log2-transformed units.

### Statistical analysis

All the data and calculated areas under the curve (AUCs) were compared between groups using the Kruskal–Wallis test followed by Holm’s post hoc pairwise comparisons (p < 0.05).

The estimation of the transmission parameters was based on a SEIR model, where each individual was considered according to the virological results as Susceptible (uninfected), Exposed (infected without virus excretion), Infectious (infected with virus excretion) or Recovered (protected without a role in the infectious process). The duration of the latency period (representing the time taken for an infected pig to become infectious, expressed in days) and the daily transmission rate (the number of pigs infected by one infectious pig per day) of the virus were estimated by Bayesian inference using the Metropolis‒Hastings algorithm. Briefly, denoting ${\varvec{p}}_{\varvec{i}}=\varvec{e}\varvec{x}\varvec{p}\left(-{\varvec{d}}_{\varvec{i}}\cdot \varvec{\beta }\cdot {\varvec{\pi }}_{\varvec{i}}\right)$ as the probability that a susceptible pig could escape infection on a time interval ${\varvec{D}}_{\varvec{i}}=[{\varvec{t}}_{\varvec{i}}, {\varvec{t}}_{\varvec{i}+1}]$ of duration ${\varvec{d}}_{\varvec{i}}$ during which the prevalence of infectious individuals was ${\varvec{\pi }}_{\varvec{i}}$, the contribution of contact animal $\varvec{j}$ in pen $\varvec{k}$ to the likelihood, i.e., the probability for its first positive serum sample to stand in the interval ${\varvec{D}}_{{\varvec{I}}_{\varvec{j}}}^{}=\left[{\varvec{t}}_{{\varvec{I}}_{\varvec{j}}}^{}, {\varvec{t}}_{{\varvec{I}}_{\varvec{j}}+1}^{}\right]$:

$${\varvec{L}}^{\left(\varvec{j}\right)}({\varvec{D}}_{{\varvec{I}}_{\varvec{j}}},{\varvec{\pi }}_{\varvec{w}}^{\left(\varvec{k}\right)},{\varvec{E}}^{\varvec{k}}|\varvec{\beta },\varvec{\gamma })=\sum _{\varvec{i}=1 }^{{\varvec{I}}_{\varvec{j}} }\left\{\prod _{\varvec{l}=1}^{\varvec{i}}{\varvec{p}}_{\varvec{l}-1}^{\left(\varvec{k}\right)} \left(1-{\varvec{p}}_{\varvec{i}}^{\varvec{k}}\right){\varvec{f}}_{\varvec{L}\varvec{a}\varvec{t}}({\varvec{t}}_{{\varvec{I}}_{\varvec{j}}}^{}-{\varvec{t}}_{\varvec{i}},\varvec{\gamma })\right\},$$

and

$$\varvec{L}({\varvec{D}}_{\varvec{I}},{\varvec{\pi }}_{\varvec{w}},\varvec{E}|\varvec{\beta },\varvec{\gamma })=\prod _{\varvec{j}=1}^{{\varvec{N}}_{\varvec{c}}}{\varvec{L}}^{\left(\varvec{j}\right)}({\varvec{D}}_{\varvec{I}},{\varvec{\pi }}_{\varvec{w}},\varvec{E}|\varvec{\beta },\varvec{\gamma }),$$

where ${\varvec{N}}_{\varvec{c}}$ is the total number of contact pigs.

Because the latency period is relatively short in regard to the infectious period [35], we modelled the latency assuming an exponential distribution, for which the rate parameter (**γ**) was estimated. The parameters were estimated by Bayesian inference using a Monte Carlo Markov Chain as described in Rose et al. (2015) [28]. Three independent chains were run with initial values randomly drawn from the prior distribution. A total of 50,000 iterations were performed, including 10% of the burn-in phase and a thinning interval of 10 iterations. Convergence was assessed by visual inspection and diagnostic tests (Gelman-Rubin, autocorrelation, Heidelberger).

## Data Availability

The datasets analysed during the current study are available from the corresponding author upon reasonable request.

## Abbreviations

PRRSV: porcine reproductive and respiratory virus
MLV: modified live vaccine
ID: intradermal
IM: intramuscular
PV: postvaccination
PC: postchallenge
D: day
V+: vaccinated
V-: unvaccinated
SPF: specific-pathogen free
TCID_50_: tissue culture infectious dose 50%
IFNg: interferon gamma
BAL: bronchoalveolar lavage
PBMCs: peripheral blood mononuclear cells, IFNg-SCs:interferon gamma-secreting cells
AUC: area under the curve
CMI: cell-mediated immunity, NAs:neutralizing antibodies, Ig:immunoglobulin, ORF:open reading frame
SD: standard deviation
